# Exploring the sequential accumulation of metabolic syndrome components in adults

**DOI:** 10.1038/s41598-022-19510-z

**Published:** 2022-09-23

**Authors:** Rodrigo Fernández-Verdejo, Jose E. Galgani

**Affiliations:** 1 Laboratorio de Fisiología del Ejercicio y Metabolismo (LABFEM), Escuela de Kinesiología, Facultad de Medicina, Universidad Finis Terrae, Avenida Pedro de Valdivia 1509, Providencia, Santiago, Chile; 2 Carrera de Nutrición y Dietética, Departamento de Ciencias de la Salud, Facultad de Medicina, Pontificia Universidad Católica de Chile, Santiago, Chile; 3 Departamento de Nutrición, Diabetes y Metabolismo, Facultad de Medicina, Pontificia Universidad Católica de Chile, Avenida Libertador Bernardo O’Higgins 340, Santiago, Chile

**Keywords:** Metabolic disorders, Risk factors, Epidemiology

## Abstract

The metabolic syndrome (MetS) is diagnosed upon the manifestation of ≥ 3 out of 5 specific components, regardless of their combination. The sequence through which these components accumulate may serve to identify underlying pathophysiological mechanisms and improve MetS treatment. We aimed to explore whether there is a more frequent sequence of accumulation of components in adults. The cross-sectional data of the National Health Survey of Chile 2016–2017 was analyzed. Subjects aged 18 to < 65 years, with body mass index ≥ 18.5 kg/m^2^, having all MetS components measured, and not under drug treatment were included (*n* = 1944, 60% women). MetS components were operationalized based on harmonized criteria: elevated waist circumference (≥ 91 cm for men, ≥ 83 cm for women), reduced high-density lipoprotein cholesterol (HDL-C; < 40 mg/dL for men, < 50 mg/dL for women), elevated triglycerides (≥ 150 mg/dL), elevated blood pressure (≥ 130 mmHg for systolic, or ≥ 85 mmHg for diastolic), and elevated glycemia (≥ 100 mg/dL). Subjects were grouped according to the number of components. Then, the prevalence of the observed combinations was determined. In subjects with one component, the most prevalent was waist circumference (56.7%). In subjects with two, the most prevalent combination was waist circumference and HDL-C (50.8%), while in subjects with three components was waist circumference, HDL-C, and triglycerides (54.0%). Finally, in subjects with four, the most prevalent combination was waist circumference, HDL-C, triglycerides, and blood pressure (40.8%). This pattern suggests that the most frequent accumulation sequence starts with abdominal obesity, followed by dyslipidemia, elevated blood pressure, and ultimately, dysglycemia. The factors that determine the sequence remain to be determined.

## Introduction

Metabolic syndrome (MetS) is a constellation of risk factors for developing cardiovascular disease and type 2 diabetes. Insulin resistance is considered an essential feature of MetS^1^, and was proposed to trigger other risk factors^2^. An index of insulin resistance was thus initially considered a requirement for MetS diagnosis^3^. Nowadays, diagnosis is based on harmonized criteria that do not consider any factor a requisite^4^. Subjects are considered afflicted with the MetS when manifesting ≥ 3 of the following risk factors (hereafter MetS components)^4^: [a] elevated waist circumference; [b] elevated triglycerides or under drug treatment for elevated triglycerides; [c] reduced high-density lipoprotein cholesterol (HDL-C) or under drug treatment for reduced HDL-C; [d] elevated blood pressure or under drug treatment for elevated blood pressure; and [e] elevated glycemia or under drug treatment for elevated glycemia.

Previous studies have focused on the prevalence and determinants of MetS^5,6^, and on the risk for cardiovascular disease^7–10^, diabetes^11^, other diseases^12,13^, and even all-cause mortality^8,14^. Nevertheless, the pathogenesis of MetS has been less explored. The sequence through which these components accumulate may depend on a progressive allostatic load. Allostatic load represents the level of demand on the system for maintaining homeostasis^15^. An excessive allostatic load may produce the system to fail. In the context of MetS, a positive energy balance induces abdominal obesity and abnormal circulating concentrations of fatty acids, adipokines, and cytokines^16^. These disturbances challenge the function of tissues, thus resulting in progressive failure. A hierarchical order for organ failure has been proposed depending on the organ's susceptibility^17^. Thus, different susceptibility of liver (regulating HDL-C and triglycerides), endothelium (regulating blood pressure), and pancreas (regulating glycemia) could determine the accumulation of MetS components. The increased MetS prevalence in older subjects^5,6^ supports the idea of allostatic load. The organism maintains homeostasis for a certain time, but the allostatic load eventually becomes unbearable. Whether a more frequent sequence of tissue failure exists, and whether tissue-specific aging^18^ affects such sequence, is unknown. Pathophysiological knowledge of the MetS may be useful for the early detection and treatment of susceptible subjects.

Notably, the detection of metabolic disturbances often leads to drug treatment. Denying such treatment would be unethical. It is therefore challenging to study the progression of MetS in the absence of drug treatment, i.e. its "natural history". Yet some efforts have been made. Franco et al.^19^ analyzed data from the Framingham Offspring Study cohort. They grouped MetS components into pairs and determined the order of appearance. In the overall sample, HDL-C preceded the appearance of any other component, whereas all components preceded the appearance of glycemia. Similar results were observed in men. In women, however, blood pressure preceded the appearance of any other component, thus suggesting sex-specific sequences of accumulation. These data provided evidence for the initial accumulation of MetS components. Nevertheless, subjects under drug treatment for elevated blood pressure were included, thus perhaps altering the natural history of MetS. In another report, Lin et al.^20^ analyzed cross-sectional data from the NHANES (1999–2002). In a structural equation model (adjusted for age and sex), obesity led –on one pathway– to insulin resistance and then dyslipidemia; and –on another pathway– to hypertension. But the variables considered in the model were not solely MetS components. For example, "obesity" considered both waist circumference and body mass index; and "dyslipidemia" considered HDL-C, triglycerides, and low-density lipoprotein cholesterol. The natural history of MetS thus remains unknown. Describing such a sequence of components accumulation can allow identifying specific phenotypic patterns that can optimize MetS treatment.

Herein, we aimed to explore the sequential accumulation of MetS components in adults. Using a large dataset, we determined the most prevalent combinations of MetS components in subjects manifesting one to four components. Subjects under drug treatment were excluded. Therefore, these data allowed us to gain insight into the natural history of MetS.

## Methods

### Study design and setting

The current study analyzed the National Health Survey of Chile 2016–2017. The study has an observational, analytical, and cross-sectional design. We followed the STROBE guidelines for reporting the findings (Supplementary Table 1). Although we used data from the Surveys of Health for epidemiologic surveillance by the Public Health Subsecretary of Chile, our findings do not compromise such Institution. The protocol and written informed consent for the survey were approved by the Scientific Ethics Committee of Pontificia Universidad Católica de Chile (CEC-MedUC, #16–019). Informed consent was obtained from all participants or, if participants were < 18 years old, from a parent and/or legal guardian. All procedures were conducted in accordance with the Declaration of Helsinki.

The survey was a cross-sectional household survey conducted between August 2016 and March 2017. Its methodological details have been described elsewhere^21^. The sampling method was stratified (30 strata) and multistage (first counties, then households, and finally one participant per household). In total, 6,233 subjects aged ≥ 15 years were surveyed.

### Participants

In our analyses, only subjects meeting the following eligibility criteria were considered: [a] 18 to < 65 years old; [b] body mass index ≥ 18.5 kg/m^2^; and [c] having all the MetS components measured. We excluded subjects who reported being under drug treatment for glycemia, cholesterol, or blood pressure.

### MetS components

The MetS components were assessed based on the harmonized criteria for clinical diagnosis of MetS^4^. Specifically: [a] waist circumference ≥ 91 cm for men, or ≥ 83 cm for women (cut-offs specific for Chile^22^); [b] circulating HDL-C < 40 mg/dL for men, or < 50 mg/dL for women; [c] circulating triglycerides ≥ 150 mg/dL; [d] systolic blood pressure ≥ 130 mmHg, or diastolic blood pressure ≥ 85 mmHg; and [e] glycemia ≥ 100 mg/dL. Thus, subjects were classified according to the number of MetS components, from zero to five. Subjects with ≥ 3 components are considered afflicted with the MetS^4^.

We also computed a MetS Z-score specific to our sample, following a previously described method^23^. The score integrates the difference between the actual value of each component and the cutoff for considering the component impaired. Thus, the MetS Z-score indicates how far the values of each component are from the cutoffs. This score represents a better severity index than summing up the number of components, as one component may have different severity: glycemia of 101 vs. 120 mg/dL. The higher the Z-score, the higher the severity of the disturbances. The Z-score for men was computed as: (40 − HDL-C)/12.1 + (Triglycerides − 150)/98.5 + (Glycemia − 100)/23 + (Waist circumference − 91)/12 + (Mean arterial pressure − 100)/11. The Z-score for women was computed as: (50 − HDL-C)/13.1 + (Triglycerides − 150)/98.5 + (Glycemia − 100)/23 + (Waist circumference − 83)/13.6 + (Mean arterial pressure − 100)/11. Mean arterial pressure was computed as: Diastolic blood pressure + (Systolic blood pressure − Diastolic blood pressure)/3.

### Data collection

Nurses obtained clinical and anthropometric measurements using standard procedures, as described^21^. Blood pressure was measured in triplicate after 5 min of rest (Omron 7200 device). The mean of the triplicates was considered for analyses. Waist circumference was measured at the middle point between the last rib and the top of the iliac crest using a plastic tape. Questionnaires were used to identify subjects under drug treatments, and those with chronic diseases (diabetes, cancer, liver disease, kidney disease). Questionnaires were also used to classify subjects as never smokers or current/former smokers, and to identify subjects with risky alcohol consumption (Alcohol Use Disorders Identification Test, short version^24^). Enzymatic assays were used to determine fasting serum concentrations of glucose, triglycerides, and HDL-C.

### Statistical analyses

All continuous variables were non-normally distributed according to the Kolmogorov–Smirnov test. Therefore, data were presented as median [25th percentile, 75th percentile] or frequencies. Independent-samples Kruskal–Wallis with pairwise *post-hoc* tests adjusted for multiple comparisons were used to compare continuous variables between groups. Pearson Chi-Square was used to analyze the association between categorical variables. IBM^®^ SPSS^®^ Statistics version 27 was used for analyses. *P* < 0.05 was considered statistically significant.

For the main analysis, we grouped all subjects according to the number of MetS components: zero, one, two, three, four, or five components. Within each group, subjects manifested different combinations of MetS components, except for the groups with zero or all components (this last group only has one combination). For example, in subjects with two components, there were subjects with either: waist circumference and HDL-C; or HDL-C and triglycerides; or triglycerides and blood pressure; and so on. We calculated the prevalence of each combination of components within each group. Then, we identified the most prevalent combinations in subjects manifesting one, two, three, and four components. This allowed us to explore the most frequent sequence of accumulation of components. We also compared the MetS Z-score of each combination of components to identify the most severe combination.

The main analysis was repeated stratified by sex (men, women), and also stratified by age quartiles (Q1: 18–28 years; Q2: > 28–38 years; Q3: > 38–50 years; Q4: > 50– < 65 years). Smoking^25,26^, alcohol consumption^27,28^, and certain chronic diseases (e.g. some cancers) can directly influence some MetS components, independently of MetS development. In sensitivity analyses, we therefore repeated the main analysis excluding current/former smokers, subjects with risky alcohol consumption, and those with chronic diseases.

## Results

### General characteristics of the subjects

**Figure 1:**
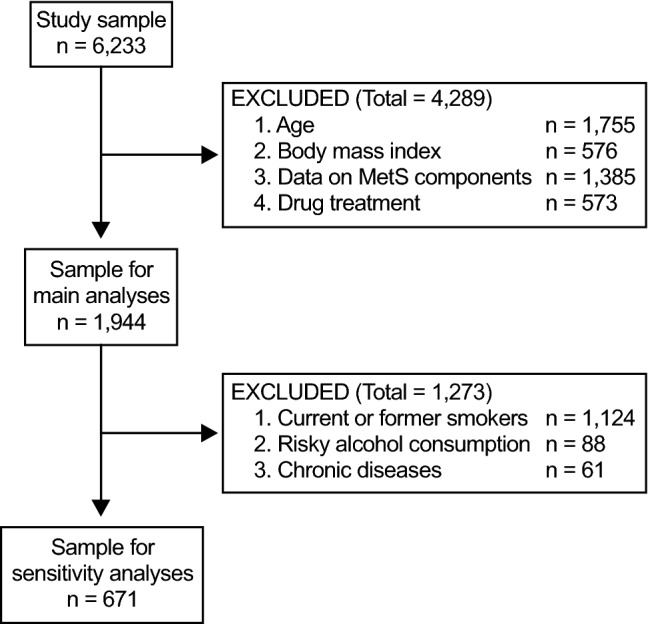
Flow diagram for the selection of subjects.

Figure 1 shows the flow diagram for the selection of subjects. After considering the eligibility and exclusion criteria, the main analysis included 1,944 subjects. Table 1 shows the characteristics of all these subjects together and by sex.

**Table 1:** Characteristics of the subjects.

*n*	All	Men	Women
	1944	768	1176
Age, years	38.0 [28.0, 50.0]	39.0 [27.0, 51.0]	38.0 [28.0, 50.0]
Weight, kg	73.0 [64.0, 84.0]	79.5 [70.2, 89.6]	68.5 [61.3, 79.1]
Height, m	1.61 [1.55, 1.68]	1.69 [1.65, 1.73]	1.57 [1.52, 1.61]
Body mass index, kg/m^2^	27.8 [25.0, 31.4]	27.5 [24.8, 30.9]	28.0 [25.0, 32.0]
Metabolic syndrome Z-score	− 1.38 [− 3.12, 0.47]	− 0.98 [− 2.97, 0.93]	− 1.64 [− 3.33, 0.18]
**Metabolic syndrome components**			
*Waist circumference, cm*	91.2 [83.0, 100.3]	94.1 [85.6, 102.0]	89.5 [81.7, 99.0]
*Mean arterial pressure, mmHg*	87.4 [80.9, 94.9]	91.9 [85.0, 99.0]	84.7 [78.8, 92.0]
*Systolic blood pressure, mmHg*	116.7 [108.0, 126.6]	123.0 [115.3, 131.7]	112.2 [104.7, 121.3]
*Diastolic blood pressure, mmHg*	72.7 [66.7, 79.6]	76.0 [69.3, 83.3]	71.0 [65.0, 77.0]
*Triglycerides, mg/dL*	117.9 [81.0, 171.0]	131.0 [94.0, 200.0]	106.5 [75.0, 157.0]
*HDL-cholesterol, mg/dL*	46.0 [38.0, 55.0]	42.0 [36.0, 50.0]	48.0 [41.0, 57.0]
*Glucose, mg/dL*	89.0 [84.0, 95.0]	91.0 [86.0, 97.0]	87.0 [82.0, 93.0]

Data are frequency, or median [25th percentile, 75th percentile].

### Prevalence of combinations of MetS components

Table 2 shows the main characteristics of all subjects grouped by the number of MetS components. About half of the subjects had one or two MetS components. Only 2.8% of subjects had all components. In subjects with one or two components, women represented a larger proportion (> 65%) compared to the other groups. Age and body mass index tended to increase from subjects with zero components to those with five components. Similar patterns were observed in men and women separately (Table 2).

**Table 2:** Characteristics of the subjects by groups of metabolic syndrome (MetS) components.

	Impaired MetS components						*P*-value*
	0	1	2	3	4	5	
**All**							
*n* (%)	338 (17.4)	490 (25.2)	526 (27.1)	367 (18.9)	169 (8.7)	54 (2.8)	–
Women, %	53.3	65.9	68.8	55.3	46.2	55.6	< 0.001
Age, years	30.0 [23.0, 42.0]^A^	37.0 [27.0, 48.0]^B^	38.0 [28.0, 50.0]^B^	41.0 [32.0, 53.0]^C^	48.0 [40.0, 55.0]^D^	51.0 [44.0, 57.3]^D^	< 0.001
Body mass index, kg/m^2^	23.4 [21.9, 25.4]^A^	26.4 [24.1, 29.4]^B^	28.8 [26.4, 32.0]^C^	30.1 [27.9, 33.1]^D^	32.3 [28.6, 35.6]^D,E^	33.6 [29.7, 36.8]^E^	< 0.001
MetS Z-score	–4.56 [–5.56, –3.62]^A^	–2.59 [–3.57, –1.77]^B^	–0.99 [–1.92, –0.12]^C^	0.60 [–0.24, 1.70]^D^	3.00 [1.80, 4.59]^E^	4.86 [3.40, 6.36]^E^	< 0.001
**Men**							
*n* (%)	158 (20.6)	167 (21.7)	164 (21.4)	164 (21.4)	91 (11.8)	24 (3.1)	–
Age, years	28.8 [21.0, 39.0]^A^	36.0 [26.0, 47.0]^B^	40.5 [30.3, 50.0]^B^	41.0 [31.0, 54.0]^B,C^	48.0 [39.0, 56.0]^C,D^	53.5 [46.0, 58.8]^D^	< 0.001
Body mass index, kg/m^2^	23.7 [22.1, 25.7]^A^	26.1 [24.3, 29.4]^B^	28.2 [25.7, 31.0]^C^	29.5 [27.1, 31.6]^C,D^	31.2 [28.8, 35.1]^D^	33.1 [28.1, 34.6]^D^	< 0.001
MetS Z-score	–4.22 [–5.39, –3.38]^A^	–2.31 [–3.01, –1.51]^B^	–0.72 [–1.65, –0.02]^C^	0.75 [–0.16, 1.92]^D^	3.09 [1.82, 4.68]^E^	5.08 [2.88, 6.24]^E^	< 0.001
**Women**							
*n* (%)	180 (15.3)	323 (27.5)	362 (30.8)	203 (17.3)	78 (6.6)	30 (2.5)	–
Age, years	33.0 [24.0, 42.8]^A^	37.0 [28.0, 48.0]^A,B^	36.0 [27.0, 49.3]^B^	41.0 [32.0, 53.0]^C,E^	49.5 [40.8, 55.0]^D^	50.5 [42.8, 53.8]^D,E^	< 0.001
Body mass index, kg/m^2^	23.2 [21.6, 25.3]^A^	26.5 [24.0, 29.4]^B^	29.1 [26.7, 32.8]^C^	31.1 [28.4, 34.5]^D^	33.0 [28.5, 37.2]^D^	34.8 [30.0, 37.1]^D^	< 0.001
MetS Z-score	− 4.79 [− 5.66, − 3.75]^A^	− 2.80 [− 3.74, − 1.96]^B^	− 1.20 [− 2.10, − 0.18]^C^	0.54 [− 0.26, 1.61]^D^	2.90 [1.77, 4.50]^E^	4.77 [3.58, 7.66]^E^	< 0.001

Values are frequency (percentage), percentage, or median [25th percentile, 75th percentile]. *Pearson Chi-Square, or Independent-samples Kruskal–Wallis. Different superscripts indicate differences between groups in the pairwise post-hoc tests (*P*-value < 0.05).

**Figure 2:**
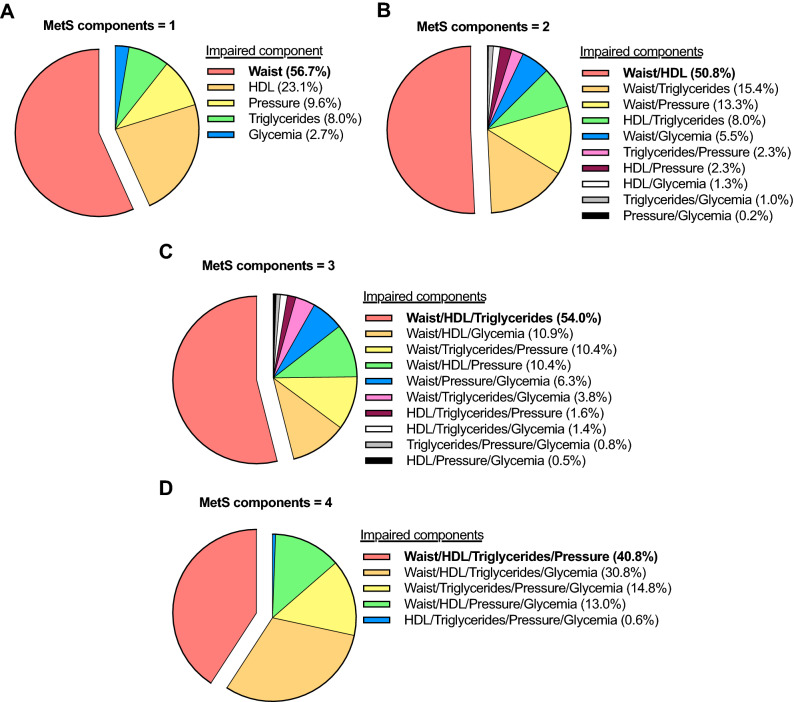
Prevalence of combinations of metabolic syndrome (MetS) components in subjects with (**A**) one, (**B**) two, (**C**) three, or (**D**) four impaired components. Waist, waist circumference; HDL, high-density lipoprotein cholesterol; Pressure, blood pressure.

Notably, in subjects with one component, the most prevalent component was waist circumference (56.7%, Fig. 2A). In subjects with two components, the most prevalent combination was waist circumference and HDL-C (50.8%, Fig. 2B). In subjects with three components, the most prevalent combination was waist circumference, HDL-C, and triglycerides (54.0%, Fig. 2C). Finally, the most prevalent combination of four components was waist circumference, HDL-C, triglycerides, and blood pressure (40.8%, Fig. 2D).

### Severity of combinations of MetS components

Table 2 shows that the higher the number of components, the higher the MetS Z-score. Supplementary Table 2 compares the MetS Z-score in subjects with the same number of components but different combinations. In subjects with one component, those with blood pressure had higher MetS Z-score than those with HDL-C or glycemia. Also, subjects with waist circumference had higher MetS Z-score than those with HDL-C or glycemia. Subjects with two to four components had similar MetS Z-score regardless of the combination.

### Prevalence of combinations of MetS components by sex

In men, the results were similar to those obtained when including all subjects, i.e. waist circumference, HDL-C, triglycerides, and blood pressure (Supplementary Fig. 1). Women only differed in the most prevalent combination of four components: waist circumference, HDL-C, triglycerides, and glycemia (47.4%). The following most prevalent combination was waist circumference, HDL-C, triglycerides, and blood pressure (37.2%, Supplementary Fig. 2).

### Prevalence of combinations of MetS components by age

Table 3 shows the main characteristics of the subjects by age quartiles. There were similar proportions of women among quartiles. Body mass index was lower in Q1 than in the other quartiles. MetS Z-score tended to increase from Q1 to Q4.

**Table 3:** Characteristics of the subjects by age quartile.

	Age quartile (years)				*P*-value*
	18–28	> 28–38	> 38–50	> 50– < 65	
*n* (%)	514 (26.4)	461 (23.7)	488 (25.1)	481 (24.7)	–
Women, %	57.8	65.1	60.5	59.0	0.109
Age, years	23.0 [20.0, 26.0]^A^	34.0 [31.0, 36.0]^B^	44.0 [42.0, 47.0]^C^	56.0 [53.0, 60.0]^D^	< 0.001
Body mass index, kg/m^2^	26.2 [23.3, 30.0]^A^	28.2 [25.4, 32.3]^B^	28.5 [25.6, 32.0]^B^	28.0 [25.5, 31.3]^B^	< 0.001
MetS Z-score	–2.70 [–4.16, –0.88]^A^	–1.46 [–3.25, 0.32]^B^	–0.98 [–2.48, 1.07]^C^	–0.44 [–2.25, 1.74]^C^	< 0.001

Values are frequency (percentage), percentage, or median [25th percentile, 75th percentile]. *Pearson Chi-Square, or Independent-samples Kruskal–Wallis. Different superscripts indicate differences between groups in the pairwise post-hoc tests (*P*-value < 0.05).

**Figure 3:**
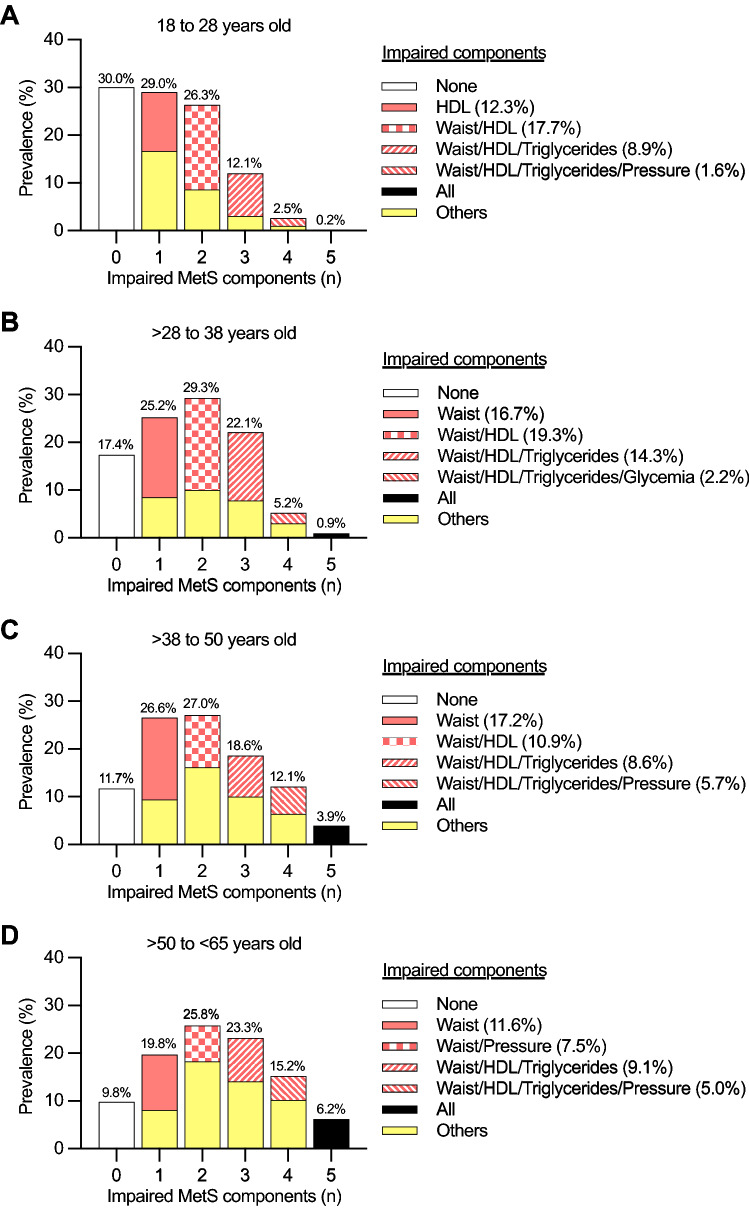
Prevalence of subjects with different number of metabolic syndrome (MetS) components by age. The most prevalent combination of MetS components is highlighted in subjects with one to four components. All percentages were calculated relative to the total subjects within each age group. Waist, waist circumference; HDL, high-density lipoprotein cholesterol; Pressure, blood pressure; Others, other combinations of MetS components.

Figure 3 summarizes the most prevalent combinations of MetS components according to the number of components. The prevalence of subjects with zero components progressively decreased from Q1 to Q4 (Q1 30.0%, Q2 17.4%, Q3 11.7%, Q4 9.8%). In contrast, the prevalence of subjects with all components progressively increased from Q1 to Q4 (Q1 0.2%, Q2 0.9%, Q3 3.9%, Q4 6.2%). So did the prevalence of subjects afflicted with the MetS (Q1 14.8%, Q2 28.2%, Q3 34.6%, Q4 44.7%).

In Q2 to Q4, the most prevalent component in subjects with one component was waist circumference (Fig. 3B–D). In Q1 to Q3, the most prevalent combination in subjects with two components was waist circumference and HDL-C (Fig. 3A–C). In all quartiles, the most prevalent combination in subjects with three components was waist circumference, HDL-C, and triglycerides (Fig. 3A–D). Finally, in Q1, Q3, and Q4, the most prevalent combination in subjects with four components was waist circumference, HDL-C, triglycerides, and blood pressure (Fig. 3A, C, and D).

### Sensitivity analyses

After excluding current/former smokers, subjects with risky alcohol consumption, and those with chronic diseases, 671 subjects remained (Fig. 1): 128 (19.1%) with zero components; 175 (26.1%) with one component; 188 (28.0%) with two components; 118 (17.6%) with three components; 43 (6.4%) with four components; and 19 (2.8%) with all components. We observed the same trends in this sample as in the main analysis. In subjects with one component, the most prevalent component was waist circumference (54.9%, Supplementary Fig. 3A). The most prevalent combination in subjects with two components was waist circumference and HDL-C (61.7%, Supplementary Fig. 3B). For three components, the most prevalent combination was waist circumference, HDL-C, and triglycerides (49.2%, Supplementary Fig. 3C). Finally, in subjects with four components, the most prevalent combination was waist circumference, HDL-C, triglycerides, and blood pressure (39.5%, Supplementary Fig. 3D).

## Discussion

The MetS is diagnosed based on accumulating at least three out of five components. Whether components accumulate randomly or follow a particular sequence is unknown. We explored the sequential accumulation of MetS components by cross-sectionally analyzing data from adults. To that end, we determined the most prevalent combinations of MetS components in subjects manifesting one, two, three, or four components. We found that the most prevalent component in subjects with one component was waist circumference. Then, waist circumference was combined with HDL-C in subjects with two components. Waist circumference and HDL-C were then combined with triglycerides in subjects with three components. Finally, waist circumference, HDL-C, and triglycerides were combined with blood pressure in subjects with four components. This pattern suggests that the most frequent sequence of accumulation of MetS components was: [i] elevated waist circumference, [ii] reduced HDL-C, [iii] elevated triglycerides, [iv] elevated blood pressure, and [v] elevated glucose. These data may help identify subjects developing MetS, and potentially design stage-specific therapies.

The MetS includes several risk factors such as insulin resistance, abdominal obesity, chronic inflammation, dyslipidemia, among others^29^. But for clinical diagnostic, only five simple components are considered^4^. Visceral obesity—manifested as elevated waist circumference—has been proposed to impair lipid metabolism, adipokine profile, and inflamation^16^. These disturbances would then affect the liver and endothelium, thus resulting in the clinical manifestation of MetS^16^. Based on this model, waist circumference should be the first MetS component manifested. Mathematical modeling of cross-sectional data supports this idea^20^. Although previous longitudinal data suggested HDL-C as the first component, waist circumference was the most strongly associated with MetS development (odds ratio [95%CI]: 4.76 [3.78–5.98])^19^. In agreement, we found that in subjects with one component, waist circumference was the most prevalent (56.7%), followed by HDL-C (23.1%). And in subjects with two components, waist circumference and HDL-C was the most prevalent combination (50.8%). This supports the idea that the most frequent progression of MetS begins with intra-abdominal fat expansion that subsequently impairs liver function (major regulator of HDL-C and triglycerides). Indeed, the most frequent combination (54.0%) in subjects with three impaired components was waist circumference, HDL-C, and triglycerides. Such finding was observed in all our analyses (sex-stratified, age-stratified, sensitivity). This suggests that strategies to prevent the MetS should target visceral fat expansion. To that end, physical activity appears as an alternative. The level of physical activity associates inversely with waist circumference^30^, and exercise training effectively reduces visceral fat^31^. In subjects with visceral obesity and an already impaired liver function, pharmacotherapy for dyslipidemia may prevent further progression.

The allostatic load could explain the sequential accumulation of MetS components. Theoretically, organs will progressively fail as allostatic load accumulates. The most susceptible organs would fail first, thus resulting in specific clinical manifestations^17^. We found that HDL-C and triglycerides were the next MetS components manifested after waist circumference. Thus, the liver appears to be the most susceptible organ to the allostatic load. Circulating glucose was the last component to be impaired in the overall sample and men. This finding suggested a lower susceptibility of the pancreas, which seems to overcome insulin resistance by increasing insulin secretion^1^. The capacity of the pancreas may have evolved given the preponderant role of circulating glucose as a fuel for tissues. The longitudinal data by Franco et al.^19^ support the relevance of glycemia over other MetS components. Therein, when components were grouped in pairs, glycemia never appeared before the other component in the pair^19^. Note that blood pressure seemed to be the last component impaired in our women (followed by glycemia). This observation is opposite to the sex-stratified analyses by Franco et al.^19^, wherein blood pressure appeared before any other component^19^. Several factors may explain the discrepancy between our findings and those by Franco et al.^19^ (e.g. sample characteristics, study design). Yet both studies suggest sex differences in the sequence of accumulation of MetS components. These differences could determine different rates of MetS development, thus explaining the different metabolic risk between sexes^6^. Future studies should test this hypothesis.

Previous data demonstrate that the prevalence of MetS increases as people age^5,6^. This is expected considering that older people have had more time to accumulate MetS components. Also, subjects may initially stand the allostatic load, but this should become unbearable in time. Our current data support these ideas. We observed a trend of increased age in subjects manifesting from zero to five components. Also, from Q1 to Q4 of age, the prevalence of MetS increased, whereas the prevalence of zero components decreased. In all age quartiles, the accumulation sequence of MetS components showed minor differences compared to the main analysis. For example, HDL-C seemed to be the first component among 18–28 years-old subjects. These results suggest age-specific variations in the sequential accumulation of MetS components. Organ susceptibility to allostatic load may vary in time due to tissue-specific aging^18^. Future studies are required to test these ideas.

We have suggested the most frequent sequence of accumulation of MetS components. Nevertheless, several other sequences may exist. The MetS is a heterogeneous and complex syndrome^17^. Our results suggest that about half of the subjects manifest the same accumulation sequence from one (waist circumference, 56.7%), to two (waist circumference and HDL-C, 50.8%), to three (waist circumference, HDL-C, and triglycerides; 54.0%) components. In turn, 40.8% of the subjects manifest the same accumulation of four components (waist circumference, HDL-C, triglycerides, and blood pressure). Nevertheless, half of the subjects would follow other sequences. An important aspect is whether the most prevalent sequence is also the most severe manifestation of MetS, or determines higher health risk. We computed a sample-specific MetS Z-score to assess severity, as previously done^23^. In subjects with one component, the impairment of waist circumference or blood pressure was more severe than the impairment of HDL-C or glycemia. No other differences in severity were observed. A previous longitudinal study showed that the triad of waist circumference, blood pressure, and glucose was associated with the highest risk for cardiovascular disease and mortality ^19^. Yet only 6.3% of our subjects manifested this triad.

The major strength of our study is the large sample size, with a wide age range, from a population-based survey. This allowed us to conduct stratified and sensitivity analyses that confirmed the robustness of the results. Yet certain limitations need to be mentioned. First, the proposed accumulation sequence of MetS components is based on cross-sectional data. This design allowed us to explore—but not demonstrate—the natural history of MetS. Second, the sample size progressively decreased from subjects with two to five MetS components. This may weaken the findings in subjects with four components. Nevertheless, note that > 70% of subjects with four MetS components manifested either of two combinations of MetS components. These two combinations should therefore be the most relevant ones. Third, early prescription of some drugs (e.g. metformin) in clinical settings may have impacted our findings. For example, subjects who manifested glycemia as the first component in their lives, and were thus prescribed metformin, were excluded from our analyses. This could explain that glycemia was the last MetS component to appear. Finally, we analyzed the National Health Survey of Chile using population-specific cutoffs for waist circumference^5,22^. Whether the same findings would apply to other populations is unknown.

In conclusion, we have suggested the existence of a natural and highly frequent development of MetS in adults. In about half of the subjects, MetS appears to develop following an accumulation of abdominal fat that subsequently leads to liver dysfunction. Also, the organism seems to preserve glycemia within the normal range as long as possible. The accumulation sequence does not seem to influence the severity of MetS. Our findings are a first approximation to understanding the natural history of MetS. Nevertheless, they should be confirmed in longitudinal studies. Future studies should also explore genetic and environmental factors underlying these phenotypic sequences, where specific lifestyle interventions may be more effective in treating them.

## Supplementary Information

Supplementary Information.

## Data Availability

The database from the National Health Survey of Chile 2016–2017 is publicly available at: http://epi.minsal.cl/. Rodrigo Fernández-Verdejo (rodrigofernandez@uft.cl) should be contacted to request the data from this study.
