# Supplementary material for: Exploring the sequential accumulation of metabolic syndrome components in adults
Source: Sci Rep. 2022 Sep 23;12:15925. doi: 10.1038/s41598-022-19510-z (PMC9508087; doi:10.1038/s41598-022-19510-z)
Supplement: Supplementary file 1 — Supplementary Information. [file 41598_2022_19510_MOESM1_ESM.docx]

**Supplementary material**

**Supplementary Table 1.** STROBE Statement—Checklist of items that should be included in reports of ***cross-sectional studies***

|  | Item No | Recommendation |  | Location |
| --- | --- | --- | --- | --- |
| **Title and abstract** | 1 | (*a*) Indicate the study’s design with a commonly used term in the title or the abstract |  | Abstract |
|  |  | (*b*) Provide in the abstract an informative and balanced summary of what was done and what was found |  |  |
| Introduction | | |  |  |
| Background/rationale | 2 | Explain the scientific background and rationale for the investigation being reported |  | Introduction, 1st to 3rd paragraphs |
| Objectives | 3 | State specific objectives, including any prespecified hypotheses |  | Introduction, 4th paragraph |
| Methods | | |  |  |
| Study design | 4 | Present key elements of study design early in the paper |  | Methods, Study design and setting |
| Setting | 5 | Describe the setting, locations, and relevant dates, including periods of recruitment, exposure, follow-up, and data collection |  |  |
| Participants | 6 | (*a*) Give the eligibility criteria, and the sources and methods of selection of participants |  | Methods, Participants |
| Variables | 7 | Clearly define all outcomes, exposures, predictors, potential confounders, and effect modifiers. Give diagnostic criteria, if applicable |  | Methods, Data collection, and MetS components |
| Data sources/ measurement | 8* | For each variable of interest, give sources of data and details of methods of assessment (measurement). Describe comparability of assessment methods if there is more than one group |  |  |
| Bias | 9 | Describe any efforts to address potential sources of bias |  | Methods, Statistical analyses |
| Study size | 10 | Explain how the study size was arrived at |  | Results, General characteristics of the subjects |
| Quantitative variables | 11 | Explain how quantitative variables were handled in the analyses. If applicable, describe which groupings were chosen and why |  | Methods, MetS components, and Statistical analyses |
| Statistical methods | 12 | (*a*) Describe all statistical methods, including those used to control for confounding |  | Methods, Statistical analyses |
|  |  | (*b*) Describe any methods used to examine subgroups and interactions |  |  |
|  |  | (*c*) Explain how missing data were addressed |  | - |
|  |  | (*d*) If applicable, describe analytical methods taking account of sampling strategy |  | - |
|  |  | (*e*) Describe any sensitivity analyses |  | Methods, Statistical analyses |
| Results | | |  |  |
| Participants | 13* | (a) Report numbers of individuals at each stage of study—eg numbers potentially eligible, examined for eligibility, confirmed eligible, included in the study, completing follow-up, and analysed |  | Results, General characteristics of the subjects |
|  |  | (b) Give reasons for non-participation at each stage |  |  |
|  |  | (c) Consider use of a flow diagram |  | Figure 1 |
| Descriptive data | 14* | (a) Give characteristics of study participants (eg demographic, clinical, social) and information on exposures and potential confounders |  | Results, General characteristics of the subjects, and Table 1 |
|  |  | (b) Indicate number of participants with missing data for each variable of interest |  | - |
| Outcome data | 15* | Report numbers of outcome events or summary measures |  | Results, Prevalence of combinations of MetS components, and Table 2 |
| Main results | 16 | (*a*) Give unadjusted estimates and, if applicable, confounder-adjusted estimates and their precision (eg, 95% confidence interval). Make clear which confounders were adjusted for and why they were included |  | - |
|  |  | (*b*) Report category boundaries when continuous variables were categorized |  | Results, Prevalence of combinations of MetS components by age, and Table 3 |
|  |  | (*c*) If relevant, consider translating estimates of relative risk into absolute risk for a meaningful time period |  | - |
| Other analyses | 17 | Report other analyses done—eg analyses of subgroups and interactions, and sensitivity analyses |  | Results, Sensitivity analyses |
| Discussion | | |  |  |
| Key results | 18 | Summarise key results with reference to study objectives |  | Discussion, 1st paragraph |
| Limitations | 19 | Discuss limitations of the study, taking into account sources of potential bias or imprecision. Discuss both direction and magnitude of any potential bias |  | Discussion, 6th paragraph |
| Interpretation | 20 | Give a cautious overall interpretation of results considering objectives, limitations, multiplicity of analyses, results from similar studies, and other relevant evidence |  | Discussion, 7th paragraph |
| Generalisability | 21 | Discuss the generalisability (external validity) of the study results |  | Discussion, 6th paragraph |
| Other information | | |  |  |
| Funding | 22 | Give the source of funding and the role of the funders for the present study and, if applicable, for the original study on which the present article is based |  | Funding |

*Give information separately for exposed and unexposed groups.

**Note:** An Explanation and Elaboration article discusses each checklist item and gives methodological background and published examples of transparent reporting. The STROBE checklist is best used in conjunction with this article (freely available on the Web sites of PLoS Medicine at http://www.plosmedicine.org/, Annals of Internal Medicine at http://www.annals.org/, and Epidemiology at http://www.epidem.com/). Information on the STROBE Initiative is available at www.strobe-statement.org.

| **Supplementary Table 2.** Metabolic syndrome (MetS) Z-score by groups of MetS components. | | | | |
| --- | --- | --- | --- | --- |
| Impaired MetS components | Median | 25th percentile | 75th percentile | n |
| MetS components = 1* |  |  |  |  |
| *Pressure^A^* | -2.31 | -3.65 | -1.00 | 47 |
| *Waist^A^* | -2.47 | -3.38 | -1.65 | 278 |
| *HDL^B^* | -2.86 | -3.88 | -2.19 | 113 |
| *Triglycerides^A,B^* | -2.93 | -3.99 | -1.82 | 39 |
| *Glycemia^B^* | -3.77 | -4.35 | -2.92 | 13 |
| MetS components = 2 |  |  |  |  |
| *Triglycerides‎/Pressure* | -0.28 | -1.02 | 0.49 | 12 |
| *Waist/Triglycerides‎* | -0.78 | -1.80 | 0.02 | 81 |
| *Waist‎/Pressure* | -0.86 | -1.66 | 0.10 | 70 |
| *Waist/Glycemia‎* | -0.94 | -2.12 | 0.24 | 29 |
| *HDL/Triglycerides‎* | -1.07 | -2.09 | -0.17 | 42 |
| *Waist/HDL‎* | -1.07 | -2.05 | -0.20 | 267 |
| *HDL‎/Pressure* | -1.22 | -1.25 | -0.30 | 12 |
| *Pressure/Glycemia‎* | -1.74 | -1.74 | -1.74 | 1 |
| *Triglycerides‎/Glycemia* | -1.78 | -1.87 | 0.12 | 5 |
| *HDL/Glycemia‎* | -2.04 | -2.61 | -1.92 | 7 |
| MetS components = 3* |  |  |  |  |
| *HDL‎/Pressure/Glycemia‎* | 1.99 | -0.14 | - | 2 |
| *Waist‎/HDL‎/Pressure* | 0.98 | -0.03 | 2.00 | 38 |
| *Triglycerides‎/Pressure‎/Glycemia* | 0.85 | -0.62 | - | 3 |
| *Waist/HDL‎/Triglycerides‎* | 0.84 | -0.08 | 1.87 | 198 |
| *HDL/Triglycerides‎/Glycemia‎* | 0.47 | -0.98 | 3.57 | 5 |
| *Waist/HDL‎/Glycemia‎* | 0.38 | -0.49 | 1.50 | 40 |
| *Waist/Triglycerides‎/Glycemia‎* | 0.33 | -0.31 | 1.79 | 14 |
| *Waist‎/Triglycerides‎/Pressure* | 0.23 | -0.55 | 1.78 | 38 |
| *HDL‎/Triglycerides‎/Pressure* | 0.03 | -0.23 | 0.22 | 6 |
| *Waist‎/Pressure/Glycemia‎* | -0.11 | -1.14 | 0.76 | 23 |
| MetS components = 4 |  |  |  |  |
| *HDL‎/Triglycerides‎‎/Pressure/Glycemia* | 5.75 | 5.75 | 5.75 | 1 |
| *Waist‎/HDL‎/Pressure/Glycemia‎* | 3.37 | 1.81 | 4.59 | 22 |
| *Waist/HDL‎/Triglycerides‎/Glycemia‎* | 3.17 | 1.16 | 4.79 | 52 |
| *Waist‎/Triglycerides/Pressure‎/Glycemia‎* | 2.91 | 1.83 | 4.84 | 25 |
| *Waist‎/HDL‎/Triglycerides‎/Pressure* | 2.87 | 1.95 | 3.94 | 69 |
| **P* < 0.05 for independent-samples Kruskal-Wallis, indicating differences in MetS Z-score between combinations of impaired components. Different superscripts indicate differences between groups in the pairwise post-hoc tests (P-value < 0.05). Waist, waist circumference; HDL, high-density lipoprotein cholesterol; Pressure, blood pressure. | | | | |

**Supplementary Figure 1.** Prevalence of combinations of metabolic syndrome (MetS) components in men with (A) one, (B) two, (C) three, or (D) four components. Waist, waist circumference; HDL, high-density lipoprotein cholesterol; Pressure, blood pressure.

**Supplementary Figure 2.** Prevalence of combinations of metabolic syndrome (MetS) components in women with (A) one, (B) two, (C) three, or (D) four components. Waist, waist circumference; HDL, high-density lipoprotein cholesterol; Pressure, blood pressure.

**Supplementary Figure 3.** Prevalence of combinations of metabolic syndrome (MetS) components in subjects with (A) one, (B) two, (C) three, or (D) four components. Current/former smokers, subjects with risky alcohol consumption, and those with chronic diseases were excluded. Waist, waist circumference; HDL, high-density lipoprotein cholesterol; Pressure, blood pressure.
